# Towards an Extended Cognitive Model of Moral Injury—The Role of Mental Defeat

**DOI:** 10.1111/sjop.70086

**Published:** 2026-03-02

**Authors:** Madelyn Letendre, Andrea Reinecke

**Affiliations:** ^1^ Department of Psychiatry University of Oxford Oxford UK

**Keywords:** cognitive model, mental defeat, moral injury, PTSD

## Abstract

Moral injury (MI) is a proposed syndrome that develops when someone is exposed to, participates in, or fails to prevent an action that fundamentally violates their moral code and results in maladaptive cognitions about oneself and humanity. Post‐traumatic stress disorder (PTSD) is a disorder based on maladaptive cognitions that develops following a traumatic event. Decades of PTSD research underscore that the experience of mental defeat, or the perception that one was completely defeated during the traumatic event, plays a significant role in the development and maintenance of PTSD. Less work, however, has been done to develop a cognitive model of MI and to understand how mental defeat plays a role in the underlying maladaptive cognitions. Understanding the cognitive model of MI is key to developing effective cognitive therapy. In this paper, we first examine the role of mental defeat in PTSD cognitive models and then compare the PTSD cognitive model to proposed cognitive models for MI. Based on these comparisons and overlapping symptomology, we suggest that mental defeat plays an important role in MI cognitive models.

## Key Points

- Overlaps in the proposed cognitive model of MI and PTSD, as well as MI symptoms, suggest that mental defeat plays a role in the development and maintenance of MI.
- Further research is required to distinguish areas of overlap and difference between PTSD and MI cognitive models to inform clinical treatment of MI.

## 1 Introduction

Moral Injury (MI) is a proposed syndrome (Jinkerson 2016) that develops when someone is exposed to, participates in, or fails to prevent an action that fundamentally violates their moral code and results in maladaptive cognitions about oneself and humanity (Boska and Capron 2021). Currently, MI is not considered a stand‐alone DSM‐5 diagnosis. Despite the inclusion of painful moral cognitions in the possible symptomology for post‐traumatic stress disorder (PTSD) in DSM‐5, the distressing moral thoughts must follow the experience of physical threat or injury to qualify for a PTSD diagnosis. This does not cover the full range of experiences that can lead to MI, such as a soldier witnessing the murder of a child by a fellow soldier, or a paramedic failing to save a patient due to a lack of preparation (Currier et al. 2021). Experiences of MI and psychological injury are increasing in prevalence within the military, due to a higher survival rate in combat (Boska and Capron 2021). Additionally, in military cultures, there is a strong suppression of negative emotions and an acceptable level of violence, which is innate to the profession (Murray and Ehlers 2021).

Although MI can develop following a traumatic event, like in PTSD, its etiology is based in moral danger and cognitive dissonance between what occurred and what the individual thinks should have happened, based on their moral code (Boska and Capron 2021). Additionally, individuals with MI experience higher levels of shame and guilt than those diagnosed with PTSD, as well as a lack of self‐forgiveness (Bryan et al. 2018) (see Table 1 for further symptom comparisons). MI also has overlapping symptomology with PTSD, including negative cognitions, avoidance of thinking about the event, and changes in information processing (Boska and Capron 2021; Murray and Ehlers 2021). Further, individuals who develop MI are likely to experience dissociation during the morally injurious event, which is indicative of mental defeat—a core factor in the development of PTSD and the maladaptive cognitions that perpetuate PTSD symptoms.

**TABLE 1:** Comparison of PTSD and MI Symptoms (based on work by Murray and Ehlers 2021; Williamson et al. 2021; Bonson et al. 2023; Boska and Capron 2021; Battles et al. 2021).

PTSD	Both	Moral injury
Etiology is mortal danger	Depression	Etiology is moral danger
Hyperarousal	Distorted, maladaptive cognitions	Higher experiences of guilt and shame
Flashbacks	Avoidance of triggers and social withdrawal	Spiritual or existential conflict
Memory loss	Thought rumination	Seek atonement
Nightmares	Negative thought appraisals	Challenge moral understanding
Fear and anxiety		Lack of self‐forgiveness
Mental defeat		

While MI is not included in the DSM‐5, its effects are significant. Individuals who experience an event that is morally injuring, in addition to being traumatic, are at a higher risk for suicidality and anxiety (Williamson et al. 2021). Further, MI could complicate treatment, as clinicians may focus on the traumatic aspects of an event, as opposed to the morally injurious aspects (Williamson et al. 2021). Currently, MI therapies include cognitive therapy (CT) protocols adapted from CT‐PTSD protocols. While the efficacy of this therapeutic approach has not been studied (Murray and Ehlers 2021), many clinicians are struggling to address MI within current trauma treatment paradigms (Currier et al. 2021). Thus, a cognitive model for MI, and the contribution of mental defeat, must be refined to support the development of MI treatments. In this paper, we will explore the role of mental defeat in cognitive model of PTSD, then examine proposed cognitive models for MI and posit that mental defeat is a contributing factor to the development and maintenance of MI, as in PTSD.

## 2 Mental Defeat

Mental defeat is experienced when someone feels completely defeated during an event. As opposed to mental planning or mental coping, where an individual makes plans about how to minimize physical and psychological harm to reduce the impact of the event, mental defeat is when an individual has the perception that they completely gave up (Ehlers et al. 1998). Further descriptors include feeling like they are no longer a person with free will, no longer a person with dignity, or no longer a person at all (Murata et al. 2019; Ehlers et al. 1998). A lack of mental planning indicates that the individual perceived that the situation was completely beyond their control. This is often reported among victims of sexual assault (Ehlers et al. 1998).

## 3 Post‐Traumatic Stress Disorder

### 3.1 Cognitive Model for PTSD

**FIGURE 1:**
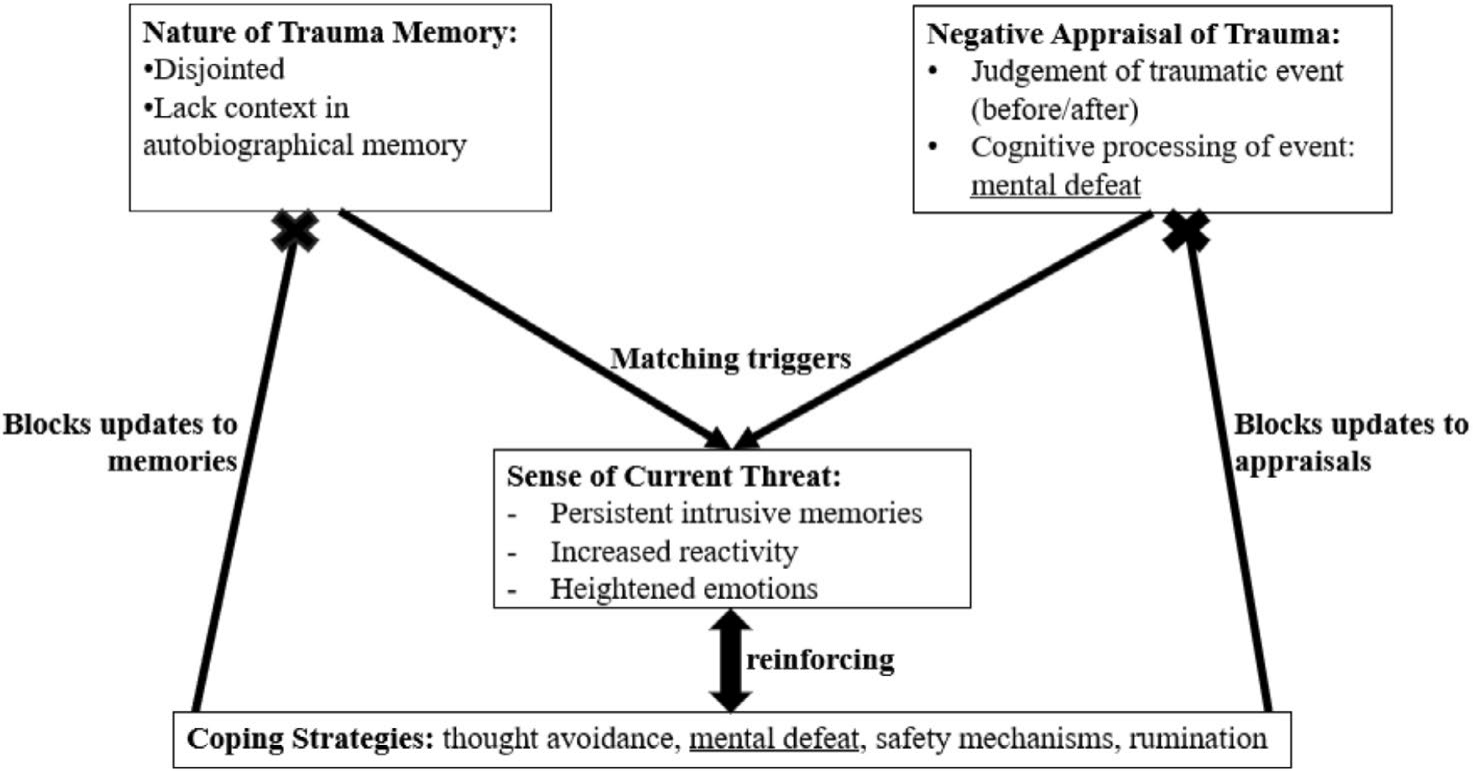
Cognitive Model of PTSD, as proposed by Ehlers and Clark (2000). Mentions of mental defeat are underlined to underscore the importance of mental defeat in the development and maintenance of PTSD, as outlined in Section 3b.

PTSD develops in reaction to a traumatic event and is characterized by persistent, involuntary re‐experiencing of the event, cognitive avoidance, negative changes in cognition and mood, and increased reactivity (American Psychiatric Association 2013). The cognitive model of PTSD is based on the characteristics of the trauma memory and excessively negative appraisals of the event, which result in the individual perceiving the traumatic event as a current threat (Boska and Capron 2021) (see Figure 1).

In PTSD, the features of the traumatic memory include disjointedness and lacking context within autobiographical memory (Ehlers et al. 2000; Kleim et al. 2008; Liberzon and Abelson 2016). Thus, memory incursions of the trauma have an intense sense of ‘nowness.’ The negative appraisals in PTSD include thoughts like “I am a weak/bad person” or “I am a worse person since this happened to me” (Duffy et al. 2013). When someone with PTSD encounters a matching trigger, often a general sensory trigger that is overly associated with the trauma, they experience a sense of current threat (Kaczkurkin et al. 2017). This sense of current threat is maintained by strategies that seek to control the threat, like thought suppression and rumination, which prevent updates to the meaning of the trauma memory and perpetuate PTSD (Wild et al. 2016; Duffy et al. 2013). To treat PTSD, clinicians must address altered cognitions that underpin the disorder (Boska and Capron 2021).

### 3.2 The Role of Mental Defeat in PTSD

Experiencing mental defeat during a traumatic event has been identified as increasing a person's risk of developing PTSD symptoms (*r* = 0.51) (Taylor et al. 2011). Another study found that mental defeat was the most impactful peritraumatic factor in the development of PTSD (Troop and Hiskey 2013; Murata et al. 2019). More so than the traumatic load or the length and frequency of traumatic events, mental defeat is the most impactful factor in the development of PTSD following trauma (Troop and Hiskey 2013). Mental defeat prevents patients from viewing traumatic events as a singular event in the past. Since the individual no longer views themselves as an individual with psychological autonomy, the traumatic event is globalized and is viewed as having current implications on events in their life (Ehlers et al. 2000). Due to the predictive power of mental defeat, it is now understood as key to the cognitive changes that underpin PTSD (Ehlers et al. 2000; Wilker et al. 2017).

Mental defeat also serves as a maintenance factor in PTSD. Negative appraisals related to mental defeat include “I am worthless,” “I deserved it,” “I am to blame for what happened” (Ehlers et al. 1998). When patients relive their trauma memories, their mental defeat cognition is perpetuated. Unless the memories are reorganized and updated, this feeling of mental defeat persists with subsequent remembering of the trauma. Thus, mental defeat is both a contributing and maintaining factor in PTSD (Troop and Hiskey 2013).

## 4 Moral Injury

### 4.1 Background on Moral Injury

MI is an increasingly prevalent area of research across multiple disciplines, including psychology, psychiatry, and social work (Griffin et al. 2019). MI is distinct from PTSD in that it develops following a potentially morally injurious event (PMIE) that violates one's moral beliefs and values. There are multiple ways a PMIE could initiate, including witnessing the event, perpetrating the event, or being betrayed by a trusted individual (Murray and Ehlers 2021; Williamson et al. 2021). For an event to be considered a PMIE, it must be a high‐stakes event that results in a transgression of established moral beliefs (Bonson et al. 2023).

Simply experiencing a PMIE does not premeditate that someone will develop MI. Moral injury develops when there is a breakdown of one's moral understanding of themselves and the world around them, leading to a degraded relationship with oneself and the rest of humanity (Bonson et al. 2023). In essence, the etiology of MI is an experience of moral danger (Boska and Capron 2021).

MI symptomology can include deeply rooted feelings of guilt and shame (‘it was all my fault’, ‘I should have done ___’), anger, and worthlessness (‘I don't matter’, ‘I'm no longer valuable’) (Williamson et al. 2021), loss of trust in self or others, and social problems (Boska and Capron 2021). The symptoms of shame and guilt are core in the development of MI (Battles et al. 2021). Thus, when a PMIE is accompanied by persistent negative appraisals of the self (Murray and Ehlers 2021), it becomes MI.

### 4.2 Moral Injury Versus PTSD

While MI is frequently comorbid with PTSD (Boska and Capron 2021), it is distinct due to differences in the causative event (MI arising from a PMIE versus PTSD arising from physical threat or injury). Additional research has distinguished differences in the cognitive maintenance and negative thought appraisals of the two disorders (altered moral understanding in MI versus hyperarousal and ‘sense of nowness’ in PTSD) (Barr et al. 2022).

These differences highlight the importance of specific cognitive models that distinguish MI from PTSD. The broad diagnosis of PTSD can overlook important cognitive differences in MI development and maintenance, preventing tailored treatment (Barr et al. 2022). Therefore, it is essential to identify where MI cognitive processes are different, as well as similar, to PTSD. Further distinctions between MI and PTSD can be found in Table 1.

### 4.3 Cognitive Model for Moral Injury

Currently, preliminary work is being carried out to develop a cognitive model of MI, largely based on symptom similarities to PTSD (Bonson et al. 2023). Cognition is key in developing morality; thus, MI will necessarily change cognition (Boska and Capron 2021). When a MI occurs, the individual develops internal conflicts with their previously held views of the justness of the world, their sense of personal esteem, and personal forgivability (Boska and Capron 2021). Across previous research, the development of MI following a PMIE rests upon two main factors.

First, the individual develops negative self‐appraisals. Rather than viewing their actions or inactions as a simple mistake, maladaptive cognitions reinforce the idea that their actions were reprehensible and cannot be forgiven. This perpetrates the symptoms of moral guilt and shame (Boska and Capron 2021). This lack of self‐forgiveness has not been shown to moderate the development of PTSD, which may help distinguish the two disorders (Griffin et al. 2019).

The second factor relates to maladaptive cognitive maintenance patterns, which prevent the integration of new information to update the individuals' understanding of the initial morally injuring event. These cognitive maintenance patterns can be broken down into three distinct categories (Murray and Ehlers 2021).

**FIGURE 2:**
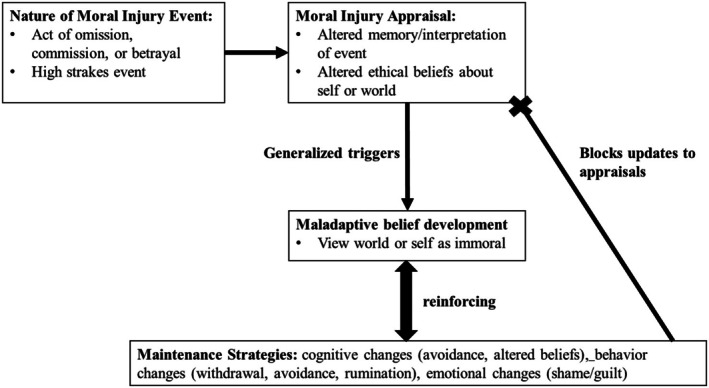
The current cognitive model of MI, based on the work of Bonson et al. (2023). In the model, a PMIE leads to negative appraisals of the event, with altered memories and altered moral understanding about the world and themselves. When the individual encounters similar situations in the future, they develop the belief that the world and/or they are immoral. This cognition is reinforced by cognitive changes, behavior changes, and emotional changes that block any updates to the appraisal of the initial PMIE.

First, cognitive changes result in the individual altering their beliefs about the surrounding world, including negative appraisals of themselves, their personal relationships, and their relationship with the world at‐large (Jamieson et al. 2020). Further, the individual avoids reevaluating the morally injurious event, preventing a re‐understanding of the event (Bonson et al. 2023). These beliefs are generalized beyond the initial PMIE and the individual processes the event as representative of their entire sense of self or sense of the world (Murray and Ehlers 2021). Second, there are negative emotional changes related to anger and shame (Bonson et al. 2023). These negative changes to mood and cognition are key risk factors for suicidal behaviors related to MI (Battles et al. 2021). Third, behavior changes include declined self‐compassion, social withdrawal, and ruminative thought patterns (Bonson et al. 2023). Especially when an individual perpetrated the PMIE, they overestimate their personal role in the event, leading to decreased self‐compassion and heightened guilt. If the event instead occurred due to a betrayal by others, the individual experiences heightened feelings of unfairness and mistrust, which results in social withdrawal (Murray and Ehlers 2021). Figure 2 outlines the basic cognitive model for MI, based on current research.

### 4.4 The Role of Mental Defeat in Moral Injury

To our knowledge, no work surrounding the current model for MI includes mental defeat as a contributing factor for the development and maintenance of MI. We propose that mental defeat plays a key role in the cognitive model of MI, based on overlaps between the cognitive models for MI and PTSD, as well as similar mental defeat and MI symptomology.

Based on previous research, experiencing mental defeat during a trauma is a key factor in the development of PTSD and the maintenance of negative self‐appraisals (Wilker et al. 2017). These negative appraisals relate to the actual traumatic event, as well as generalizations about the individual themselves (‘I am weak’, ‘I am a worse person’). As outlined in section 2b, peritraumatic mental defeat increases the likelihood of avoidance behaviors, which blocks any sort of update to the trauma memory (Wilker et al. 2017). We believe that the negative appraisals in MI may be similarly blocked from being updated due to mental defeat.

**FIGURE 3:**
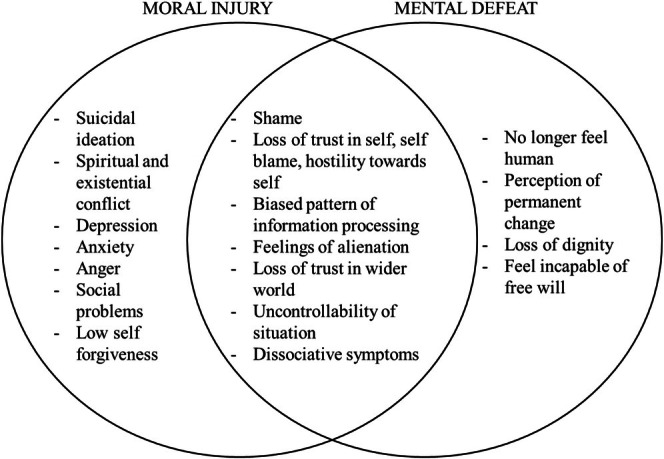
Overlapping symptoms of MI and Mental Defeat. This chart synthesizes the overlap of mental defeat and MI, supporting that mental defeat may be a contributing factor to the development of MI. Symptoms of MI are based on the research of Williamson et al. (2021); Boska and Capron (2021); Ehlers et al. (1998); Bonson et al. (2023); Murray and Ehlers (2021). Symptoms of mental defeat are based on the work of Boska and Capron (2021); Ehlers et al. (1998); Bonson et al. (2023); Murray and Ehlers (2021); Murata et al. (2019).

Evidence for mental defeat contributing to MI comes from coinciding symptoms (Wilker et al. 2017), as seen in Figure 3. Across MI and mental defeat literature, key symptoms include shame, loss of trust in oneself and humanity, and a feeling of uncontrollability/dissociation during the event (Ehlers et al. 1998; Boska and Capron 2021; Murray and Ehlers 2021; Bonson et al. 2023).

**FIGURE 4:**
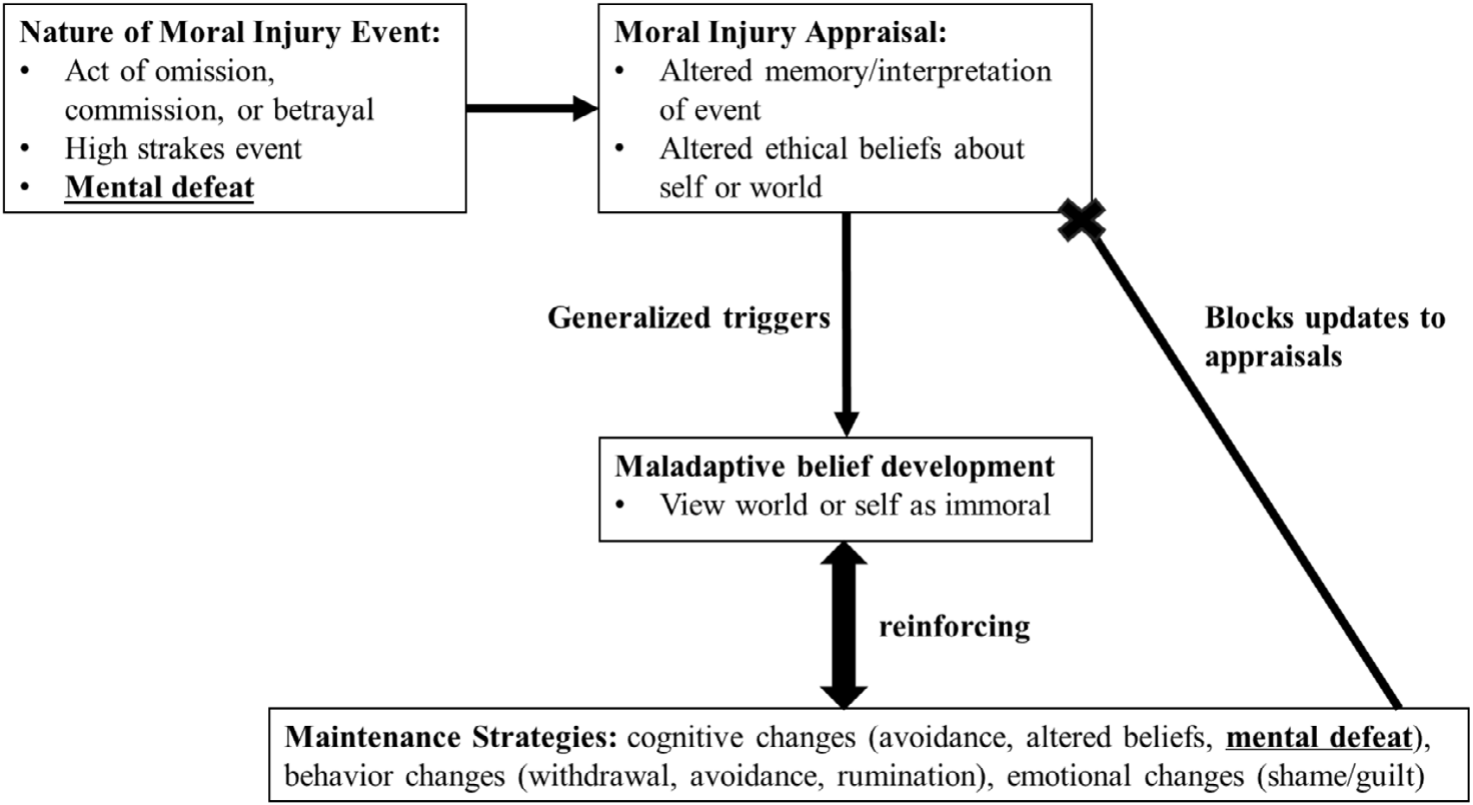
Proposed extended cognitive model for MI. This model expands on the previously proposed model (Figure 2), with the addition of mental defeat. We suggest that during the initial PMIE, mental defeat results in individuals dissociating or experiencing mental shut‐down, contributing to the development of MI. Following the development of MI, we suggest that mental defeat serves as a cognitive maintenance strategy, with the individual perceiving the MI as a permanent change to their sense of self and personhood.

During PMIEs that result in MI, patients often report dissociative, out‐of‐body experiences. Within high‐stress and highly professional careers, such as military and medical environments, individuals often distance themselves emotionally from their work, which can result in these out‐of‐body experiences (Murray and Ehlers 2021). Like the dissociative symptoms often seen in MI, mental defeat occurs when the self‐defense mechanisms are exhausted, and the individual reaches a state of dissociative shutdown. Based on the substantial overlap in symptomology between MI and mental defeat, we suggest that the experience of mental defeat during a PMIE could contribute to the development of MI (Wilker et al. 2017). An updated model of MI, with the inclusion of mental defeat, is outlined in Figure 4. To confirm this prediction, more research must be done to examine the cognitive model of MI.

## 5 Conclusion‐ Gaps and Future Study

In this paper, we suggest that one important overlap between PTSD and MI may be mental defeat. There is extensive work exploring the relationship between mental defeat and PTSD. There is limited research, however, exploring the role of mental defeat in initiating and maintaining MI. Based on previous research on both mental defeat and MI, we have found significant overlaps in symptomology, including dissociation, a sense of uncontrollability surrounding the situation, and a loss of trust in the wider world. The importance of mental defeat in the development and maintenance of MI should be further researched as mental defeat may be an important factor in the mental model for MI and would be an important consideration when developing treatments.

Additionally, work must be done to understand the overlaps between PTSD and MI (such as mental defeat) and the differences between the disorders. Overlaps between PTSD and MI can inform which PTSD therapies can be repurposed for the treatment of MI (Williamson et al. 2021). Already, aspects of cognitive therapy for PTSD have been modified to work for MI, with some success (Wilker et al. 2017). For example, imagery rescripting has been trialed to treat the symptoms of mental defeat in PTSD. This consists of imagining the traumatic experience and then visualizing an alternative course of action for the event. This thought intervention encouraged flexibility in the cognitive model of the traumatic memory and improved mental defeat scores (Murata et al. 2019). Since we propose mental defeat is an area where PTSD and MI overlap, imagery rescripting could be trialed as a treatment strategy to address mental defeat in MI.

In areas where the cognitive models for MI and PTSD differ, such as the disruption in moral values in MI, new approaches to therapy must be explored (Farnsworth et al. 2017; Murray and Ehlers 2021). While there is an overlap between the cognitive model for PTSD and the proposed cognitive model for MI, treatments for PTSD have not been sufficient in addressing PTSD in individuals who also experienced MI (Boska and Capron 2021). This may be due to clinicians focusing on the traumatic aspects of the event, rather than treating the moral injury which underpins a patient's symptomology (Williamson et al. 2021; Barr et al. 2022). Possible avenues of exploration to effectively supplement MI cognitive treatment include therapies that focus on reparations, such as Adaptive Disclosure or Acceptance and Commitment Therapy, which could be used in conjunction with CT‐PTSD (Litz et al. 2015; Lee 2018; Williamson et al. 2021). Since the thought appraisals involved in MIs are often more complex and nuanced than in PTSD, there must be more work done to establish characteristic cognitions in MI and guide therapeutic approaches (Barr et al. 2022).

This paper outlines a domain where MI and PTSD may overlap—mental defeat. However, further research must seek to understand which symptoms and cognitive patterns specifically underpin MI and distinguish the disorder from PTSD. Until research outlines a comprehensive cognitive model of MI, clinicians will be reliant on traditional trauma paradigms, which do not capture the full nuance of MI. To improve the efficacy of treatment, researchers must first develop a clearer understanding of the underlying cognitive model of MI, which requires additional research into the overlaps and divergences from PTSD (Murray and Ehlers 2021).

## Author Contributions

Madelyn Letendre is the main author, with contributions and edits by Andrea Reinecke.

## Funding

Madelyn Letendre is funded by the Rhodes Scholarship provided by the Rhodes Trust. Andrea Reinecke is funded by a University of Oxford Brain Sciences Fellowship.

## Ethics Statement

The authors have nothing to report.

## Conflicts of Interest

The authors declare no conflicts of interest.

## Data Availability

Data sharing not applicable to this article as no datasets were generated or analyzed during the current study.

## References

[sjop70086-bib-0001] American Psychiatric Association . 2013. Diagnostic and Statistical Manual of Mental Disorders. 5th ed. American Psychiatric Association. 10.1176/appi.books.9780890425596.

[sjop70086-bib-0002] Barr, N. , H. Atuel , S. Saba , and C. A. Castro . 2022. “Toward a Dual Process Model of Moral Injury and Traumatic Illness.” Frontiers in Psychiatry 13: 1–11. 10.3389/fpsyt.2022.883338/full. PMC9448886 36090367

[sjop70086-bib-0003] Battles, A. , J. Jinkerson , M. Kelley , and R. Mason . 2021. “Structural Examination of Moral Injury and PTSD and Their Associations With Suicidal Behavior Among Combat Veterans.” JCES 13: 1–14. https://jces.ua.edu/articles/68.

[sjop70086-bib-0004] Bonson, A. , D. Murphy , V. Aldridge , N. Greenberg , and V. Williamson . 2023. “Conceptualization of Moral Injury: A Socio‐Cognitive Perspective.” Journal of Military, Veteran and Family Health 9: 75–81. 10.3138/jmvfh-2022-0034. PMC13292905 42533870

[sjop70086-bib-0005] Boska, R. L. , and D. W. Capron . 2021. “Exploring the Maladaptive Cognitions of Moral Injury Within a Primarily Combat‐Trauma Military Sample.” Psychological Trauma Theory Research Practice and Policy 13: 861–868. 10.1037/tra0001071. 34435814

[sjop70086-bib-0006] Bryan, C. J. , A. O. Bryan , E. Roberge , F. R. Leifker , and D. C. Rozek . 2018. “Moral Injury, Posttraumatic Stress Disorder, and Suicidal Behavior Among National Guard Personnel.” Psychological Trauma Theory Research Practice and Policy 10: 36–45. 10.1037/tra0000290. 28581315

[sjop70086-bib-0007] Currier, J. M. , K. D. Drescher , and J. Nieuwsma . 2021. “Introduction to Moral Injury.” In Addressing Moral Injury in Clinical Practice, 3–18. American Psychological Association. 10.1037/0000204-001.

[sjop70086-bib-0008] Duffy, M. , D. Bolton , K. Gillespie , A. Ehlers , and D. M. Clark . 2013. “A Community Study of the Psychological Effects of the Omagh Car Bomb on Adults.” PLoS One 8: e76618. 10.1371/journal.pone.0076618. 24098795 PMC3787106

[sjop70086-bib-0009] Ehlers, A. , and D. M. Clark . 2000. “A Cognitive Model of Posttraumatic Stress Disorder.” Behaviour Research and Therapy 38: 319–345. https://linkinghub.elsevier.com/retrieve/pii/S0005796799001230. 10761279 10.1016/s0005-7967(99)00123-0

[sjop70086-bib-0010] Ehlers, A. , D. M. Clark , E. Dunmore , L. Jaycox , E. Meadows , and E. B. Foa . 1998. “Predicting Response to Exposure Treatment in PTSD: The Role of Mental Defeat and Alienation.” Journal of Traumatic Stress 11: 457–471. 10.1023/A:1024448511504. 9690187

[sjop70086-bib-0011] Ehlers, A. , A. Maercker , and A. Boos . 2000. “Posttraumatic Stress Disorder Following Political Imprisonment: The Role of Mental Defeat, Alienation, and Perceived Permanent Change.” Journal of Abnormal Psychology 109: 45–55. 10.1037/0021-843X.109.1.45. 10740935

[sjop70086-bib-0012] Farnsworth, J. K. , K. D. Drescher , W. Evans , and R. D. Walser . 2017. “A Functional Approach to Understanding and Treating Military‐Related Moral Injury.” Journal of Contextual Behavioral Science 6: 391–397. https://linkinghub.elsevier.com/retrieve/pii/S2212144717300601.

[sjop70086-bib-0013] Griffin, B. J. , N. Purcell , K. Burkman , et al. 2019. “Moral Injury: An Integrative Review.” Journal of Traumatic Stress 32: 350–362. https://onlinelibrary.wiley.com/doi/10.1002/jts.22362. 30688367 10.1002/jts.22362

[sjop70086-bib-0014] Jamieson, N. , M. Maple , D. Ratnarajah , and K. Usher . 2020. “Military Moral Injury: A Concept Analysis.” International Journal of Mental Health Nursing 29, no. 6: 1049–1066. 10.1111/inm.12792. 33078522

[sjop70086-bib-0029] Jinkerson, J. D. 2016. “Defining and assessing moral injury: A syndrome perspective.” Traumatology 22, no. 2: 122–130. 10.1037/trm0000069.

[sjop70086-bib-0015] Kaczkurkin, A. N. , P. C. Burton , S. M. Chazin , et al. 2017. “Neural Substrates of Overgeneralized Conditioned Fear in PTSD.” American Journal of Psychiatry 174: 125–134. 10.1176/appi.ajp.2016.15121549. 27794690 PMC7269602

[sjop70086-bib-0017] Kleim, B. , F. Wallott , and A. Ehlers . 2008. “Are Trauma Memories Disjointed From Other Autobiographical Memories in Posttraumatic Stress Disorder? An Experimental Investigation.” Behavioural and Cognitive Psychotherapy 36: 221–234 2. 21241538 10.1017/S1352465807004080 PMC2889292

[sjop70086-bib-0018] Lee, L. J. 2018. Moral Injury Reconciliation: A Practitioner's Guide for Treating Moral Injury, PTSD, Grief, and Military Sexual Trauma Through Spiritual Formation Strategies. Jessica Kingsley Publishers.

[sjop70086-bib-0020] Liberzon, I. , and J. L. Abelson . 2016. “Context Processing and the Neurobiology of Post‐Traumatic Stress Disorder.” Neuron 92: 14–30. 27710783 10.1016/j.neuron.2016.09.039 PMC5113735

[sjop70086-bib-0021] Litz, B. T. , L. Lebowitz , M. J. Gray , and W. P. Nash . 2015. Adaptive Disclosure: A New Treatment for Military Trauma, Loss, and Moral Injury. Guilford Publications.

[sjop70086-bib-0022] Murata, T. , Y. Hiramatsu , F. Yamada , et al. 2019. “Alterations of Mental Defeat and Cognitive Flexibility During Cognitive Behavioral Therapy in Patients With Major Depressive Disorder: A Single‐Arm Pilot Study.” BMC Research Notes 12: 723. 10.1186/s13104-019-4758-2. 31694691 PMC6833291

[sjop70086-bib-0023] Murray, H. , and A. Ehlers . 2021. “Cognitive Therapy for Moral Injury in Post‐Traumatic Stress Disorder.” Cognitive Behaviour Therapist 14: e8. 34191944 10.1017/S1754470X21000040 PMC7853755

[sjop70086-bib-0024] Taylor, P. J. , P. Gooding , A. M. Wood , and N. Tarrier . 2011. “The Role of Defeat and Entrapment in Depression, Anxiety, and Suicide.” Psychological Bulletin 137: 391–420. 10.1037/a0022935. 21443319

[sjop70086-bib-0025] Troop, N. A. , and S. Hiskey . 2013. “Social Defeat and PTSD Symptoms Following Trauma.” British Journal of Clinical Psychology 52: 365–379. 10.1111/bjc.12022. 24117910

[sjop70086-bib-0026] Wild, J. , K. V. Smith , E. Thompson , F. Béar , M. J. J. Lommen , and A. Ehlers . 2016. “A Prospective Study of Pre‐Trauma Risk Factors for Post‐Traumatic Stress Disorder and Depression.” Psychological Medicine 46: 2571–2582. https://www.cambridge.org/core/product/identifier/S0033291716000532/type/journal_article. 27348599 10.1017/S0033291716000532 PMC4988264

[sjop70086-bib-0027] Wilker, S. , B. Kleim , A. Geiling , A. Pfeiffer , T. Elbert , and I. T. Kolassa . 2017. “Mental Defeat and Cumulative Trauma Experiences Predict Trauma‐Related Psychopathology: Evidence From a Postconflict Population in Northern Uganda.” Clinical Psychological Science 5: 974–984. 10.1177/2167702617719946.

[sjop70086-bib-0028] Williamson, V. , D. Murphy , S. A. M. Stevelink , S. Allen , E. Jones , and N. Greenberg . 2021. “The Impact of Moral Injury on the Wellbeing of UK Military Veterans.” BMC Psychology 9: 73. 10.1186/s40359-021-00578-7. 33952352 PMC8097892

